# Developing the Chinese version of the Index of Sojourner Social Support: the roles of socio-emotional and instrumental support in internal migrant university students

**DOI:** 10.3389/fpsyg.2025.1485375

**Published:** 2025-02-12

**Authors:** Hanzhi Fu

**Affiliations:** SISU Intercultural Institute (SII), Shanghai International Studies University (SISU), Shanghai, China

**Keywords:** Index of Sojourner Social Support, socio-emotional support, instrumental support, scale validation, internal migration, university students, China

## Abstract

**Introduction:**

This study aims to validate the Index of Sojourner Social Support (ISSS)—a widely-used scale for measuring social support in cross-cultural (including cross-national and internal) migrations—in Chinese contexts among internal migrant university students and explore how such support can facilitate their psychological adjustment.

**Methods:**

One thousand six hundred ninety-two university students who migrated from all around China to the city of Shanghai participated in this study. The ISSS was translated according to strict procedures. Item analysis, confirmatory factor analysis (CFA), reliability analysis, measurement equivalence test, criterion validity test, and incremental validity test of the Chinese version of the ISSS (ISSS-C) were carried out.

**Results:**

The ISSS-C generated by this study had two dimensions (socio-emotional support and instrumental support) of 18 items. The model fit was excellent (χ^2^/df = 5.64, CFI = 0.96, TLI = 0.95, SRMR = 0.03, and RMSEA = 0.06). The McDonald's omegas for its two subscales were both 0.96. The measurement equivalence of the ISSS-C and criterion validity were also excellent. Instrumental support significantly influenced students' psychological adjustment to the host culture, partially establishing incremental validity for the scale.

**Discussion:**

The ISSS-C exhibits good psychometric properties and is appropriate for measuring social support perceived by migrant university students in Chinese cultural contexts. Further, instrumental support can assist them in psychologically adjusting well to local environments.

## 1 Introduction

Social support in general refers to the care, aid, or networks of physical and psychological assistance individuals perceive or actually receive from others to deal with stress (House, 1981; Cohen, 2004). It is a broad “meta-construct” encompassing a series of sub-constructs (Heller and Swindle, 1983; Vaux et al., 1987) that play essential roles in reducing stress and promoting physical and mental wellbeing during life-changing scenarios (Andrews et al., 1978; Dean and Ensel, 1982; Argyle, 1992; Yue et al., 2024).

During transitions to distinct cultural environments, social support has been shown to positively impact individuals' cross-cultural adaptation, which refers to the psychological outcome of individuals' higher levels of comfort, ease, or satisfaction in culturally distinct settings (Black, 1988), and also acculturation, which represents the process of managing transitions in a new cultural context (Berry, 1997). Such positive influences have been known to ameliorate stress (Adelman, 1988; Walton, 1990; McLean et al., 2023) and robustly predict psychological adjustment to local conditions (Zhang and Goodson, 2011; Bender et al., 2019).

In transitions across cultures, social support is usually made up of two components—socio-emotional support and instrumental support (Ong and Ward, 2005). Socio-emotional support concerns the assistance provided by individuals who reassure their affection and respect, offer company and consolation, and share both joys and sorrows. More often offered by people of the host culture, socio-emotional support is predictive of lower levels of depression and promotes adaptation during transitioning periods (Ramsay et al., 2007; Dawson and Samek, 2022). Instrumental support, on the other hand, entails having individuals who elucidate the local group's culture, help navigate local regulations, assist in orienting to new surroundings, and provide information regarding available options. This component has also been found to reduce the negative effects in acculturation and play a more critical role in facilitating psychological adjustment in cross-cultural migrations (Finch and Vega, 2003; Ong and Ward, 2005; Tindle et al., 2022).

Social support in intercultural transitions can be measured in either objective or subjective manners (Solomon et al., 1987). Objective social support is usually represented by received social support which concerns the supportive behaviors offered by one's network (Barrera, 1986). Subjective social support is typically measured by perceived social support (Zimet et al., 1988; Lee and Robbins, 1995), reflecting the perceived availability of social help that buffer stress during relocation (Ong and Ward, 2005). It has been shown that, compared with objective social support, subjective social support is more positively related to mental health (Prati and Pietrantoni, 2010; Yan et al., 2024).

As regards sources, social support in cross-cultural contexts comes from individuals of the same cultural background and those of the host culture. Strong connections with individuals of the same cultural group can help people adapt to local conditions well (Al-Sharideh and Goe, 1998) and support from the host network promotes their wellbeing during the acculturation processes (Searle and Ward, 1990; Zhang and Goodson, 2011; Geeraert and Demoulin, 2013; Hirai et al., 2015).

Over decades, researchers have developed numerous measurement instruments for assessing social support (e.g., Barrera et al., 1981; Flaherty et al., 1983; Procidano and Heller, 1983; Vaux et al., 1986; Zimet et al., 1988; Tracy and Whittaker, 1990; Mitchell et al., 2003; Gordon-Hollingsworth et al., 2016). However, since social support has been established as a meta-construct that can be conceptualized into different components for different purposes or situations in operation (Vaux, 1988; Vazquez-Morejon and Garcia-Boveda, 1997), different scales assess social support from highly distinct perspectives. The validity of intercultural adaption studies is often undermined when they use social support assessment tools not intended for such a purpose (Veiel, 1985; Ong and Ward, 2005). To address this problem, the Index of Sojourner Social Support (ISSS) Scale (Ong and Ward, 2005) was compiled and has soon been widely used in this regard.

The ISSS is a comprehensive instrument tailor-made for cross-cultural settings that measures individuals' perceived social support from the sources of their own and host networks using two components (socio-emotional support and instrumental support). To develop this scale, Ong and Ward (2005) initially generated a total of 64 items from existing measures and from a survey administered by themselves among expatriates in Singapore. Based on an exploratory factor analysis, content analysis, and item-total and inter-item correlation analyses of the 64 items, the original English version of the ISSS scale of 18 items and two factors were generated. Measurement invariance, construct validity, and incremental validity tests that followed further supported this two-factor configuration. They also tested the scale among international students in New Zealand. The results supported the original two-factor model though a one-factor solution (18 items) also exhibited good model fit.

Later, the ISSS was examined by other researchers in non-English-speaking societies. A validation study by Gilbert and Rhodes (2012) using a translated Spanish version of the ISSS revealed that the original two factors were not sufficiently distinct constructs. Instead, they respecified a one-factor measure containing 11 items. Rhodes et al. (2013) tested the ISSS in another Spanish-speaking community. The results showed that three items cross-loaded on both factors, undermining the scale's discriminant validity. After removing the three items, a new two-factor model of 15 items was formed and exhibited improved model fit.

Since its compilation, the ISSS has been widely adopted in studies regarding cross-cultural migrations, a considerable number of which were carried out in Chinese contexts. While a few of these studies examined sojourners' cross-national experience (e.g., Cheng et al., 2018; English et al., 2021), the overwhelming majority of them delved into internal migration situations within China (e.g., Li and Xia, 2018; Wang et al., 2018; Chen and Yang, 2022; Xiong R. et al., 2021; Xiong M. et al., 2021; Ni et al., 2016; Chen et al., 2021; Xiong et al., 2023). However, despite its wide application in China, no thorough validation study of the ISSS has ever been carried out in Chinese contexts. Most existing studies utilizing the original ISSS in China merely reported Cronbach's alpha coefficients and only one study reported satisfactory model fit. Nevertheless, given the aforementioned mixed results of factorial and discriminant validity tests of this scale in other non-English speaking cultural contexts and the fact that structures of scales concerning cross-cultural issues often change in different cultural conditions (Bücker et al., 2015; Bahar-Özvarıs et al., 2022; Fu et al., 2024), using the original ISSS not thoroughly evaluated in local contexts may undermine the validity of relevant research (Brown et al., 2015). Therefore, it is crucial to thoroughly explore the latent constructs of cross-cultural perceived social support in Chinese contexts through testing the dimensionality of the ISSS in such cultural environments, and further examine the validity and reliability of the model.

In addition, as noted, though social support in cross-cultural situations positively influences psychological adjustment—a concept focusing on emotional wellbeing, satisfaction with life, and a sense of purpose and hope (Ward and Kennedy, 1999)—and migrations within China can be regarded as cross-cultural (English and Worlton, 2017) due to the country's large size and its vast cultural diversity (Talhelm et al., 2014; Talhelm and English, 2020), no study has considered whether social support can predict psychological adjustment in intra-country migrations, even though most studies using the ISSS in Chinese contexts were conducted in such conditions.

In light of the gaps in the extant body of literature, the present study aims to conduct a thorough large-scale test and revision of the ISSS among Chinese migrant university students in order to develop and validate a Chinese version of the ISSS (ISSS-C). Furthermore, this study is also intended to find out whether social support in internal migration settings can help students adapt well psychologically to local conditions in Shanghai, a viable place for such research because this dynamic multicultural city is not only a hub for higher education where numerous universities attract applicants from all over China, but also a global economic center where, compared to other parts of the country, there are more people from different parts of China and the world seeking opportunities (Farrer, 2016; Tian and Liu, 2021). University students here have more chances of interacting with people from diverse cultural backgrounds.

## 2 Materials and methods

### 2.1 Procedure and participants

This study employed a cross-sectional design to retrieve data from university students in Shanghai using questionnaire surveys. As suggested by extant scale development and validation studies (Ong and Ward, 2005; Huang and Wen, 2021), validity tests (including criterion validity and incremental validity tests) that involve other scales are better performed on new samples to further validate the structure of the scale. Therefore, three online questionnaires were created using Questionnaire Star (http://www.wjx.cn) and distributed via QR codes. The first survey (21 items) was intended to develop the ISSS-C and evaluate its model fit, convergent validity, discriminant validity, reliability, and measurement invariance. To further test the new scale's criterion validity and incremental validity, two more surveys (31 items and 29 items) were administered with the addition of the Chinese version of the Multi-dimensional Scale of Perceived Social Support (MSPSS; Wang et al., 2017) and the Schwartz Outcome Scale (SOS-10; Fu et al., 2024), respectively, together with the ISSS-C. Data were collected via convenience sampling. Specifically, teachers of three universities located in Shanghai (a natural science university, a social science university, and a medical university) were contacted to help distribute the online questionnaires among their own students (using the same three QR codes for the three surveys) before their class sessions or in their classes' Wechat (a popular communication application in China) chat groups.

The inclusion criteria of participants were driven by two principles: they must be undergraduate or postgraduate students enrolled in academic programs of universities in Shanghai, and they must be living in Shanghai but did not live there before attending universities. The data collection of the three surveys lasted 5 months (from January to May, 2024) and according to teachers recruited for the questionnaire distribution, at the time of the data collection, there were 2,231 students enrolled in their classes and 1,982 of them participated in the current research (89% participation rate). A total of 1,692 valid questionnaires were obtained after removing different kinds of invalid ones such as those with regular answers and those that failed to respond correctly to an embedded attention check item. The samples of the three surveys contained 1,206 (48.5% female; M_age_ = 20.19), 151 (46.4% female; M_age_ = 22.18), and 335 (51.9% female; M_age_ = 21.04) participants, respectively. Details of the samples can be found in Table 1. According to Hair et al. (2018), a sample-to-variable ratio in questionnaire surveys should ideally exceed 20:1. Thus, the three samples of 1,206 respondents (40:2), 151 respondents (100:5), and 335 respondents (60:3) all surpassed the minimum required levels. All subjects participated voluntarily in this study and received a randomly drawn bonus of gratitude (one to two Yuan) by the Questionnaire Star system upon completion. All methods in the Questionnaire Star system were carried out in accordance with Personal Information Protection Law of the People's Republic of China.

**Table 1:** Background of the samples.

**Samples**		**Frequency**	**Percentage**
**Survey 1 sample (*****n*** = **1,206)**			
1. What is your age?	18–22	983	81.5
	23–27	210	17.4
	28 and above	13	1.1
2. What is your gender?	Male	621	51.1
	Female	585	48.5
3. What is the size of your hometown?	Big city	443	36.7
	Medium or small city	354	29.4
	Town or village	409	33.9
**Survey 2 sample (*****n*** = **151)**			
1. What is your age?	18–22	66	43.7
	23–27	83	55.0
	28 and above	2	1.3
2. What is your gender?	Male	81	53.6
	Female	70	46.4
3. What is the size of your hometown?	Big city	34	22.5
	Medium or small city	46	30.5
	Town or village	71	47.0
**Survey 3 sample (*****n*** = **335)**			
1. What is your age?	18–22	203	61.8
	23–27	129	37.3
	28 and above	3	0.9
2. What is your gender?	Male	161	48.1
	Female	174	51.9
3. What is the size of your hometown?	Big city	103	30.7
	Medium or small city	110	32.8
	Town or village	122	36.4

### 2.2 Measurement tools

All three surveys in the current study consisted of two segments. The first segment included demographic information regarding students' gender, age, and the size of their hometown which has been shown to have potential influences on the results of internal migration studies in China (Chen and Wong, 2022; Fu et al., 2024). The second section contained one to two instruments depending on the purpose of that survey. Specifically, the second section of the first survey contained the 18-item ISSS for developing the ISSS-C. The second section of the second survey included the ISSS-C and the Multi-dimensional Scale of Perceived Social Support (MSPSS) Chinese version in order to test the criterion validity of the ISSS-C. For the third survey, the second segment comprised the ISSS-C and the Chinese version of the Schwartz Outcome Scale (SOS-10) to test whether socio-emotional or instrumental support can predict students' psychological adjustment in internal migration settings and provide evidence for the ISSS-C's incremental validity. All items in this study were presented in Chinese. Five-point Likert-scale questions were used in all the three measures (1 = completely not applicable, 5 = completely applicable).

The 18-item Index of Sojourner Social Support (Ong and Ward, 2005) was applied to develop its Chinese counterpart. The ISSS evaluates perceived social support in intercultural contexts with two subscales—socio-emotional support (9 items; e.g., “comfort you whenever you feel homesick”) and instrumental support (9 items; e.g., “provide necessary information to help orient you to your new surroundings”). All the items in the ISSS were translated and back-translated by a local English language teacher and a native English-speaking teacher proficient in Chinese following standard procedures (Brislin, 1980); the wording was also checked for fitness in local contexts.

The Chinese version of the Multi-dimensional Scale of Perceived Social Support (Wang et al., 2017) was employed to establish criterion validity for the new ISSS-C. Similar to the ISSS-C, the Chinese version of MSPSS also measures perceived social support (but in a comprehensive manner), and is one of the most widely-used scales in this domain (Wang et al., 2017). The MSPSS Chinese version is a 12-item instrument comprising three dimensions—support from family (4 items; e.g., “my family really tries to help me”), support from friends (4 items; e.g., “I can talk about my problems with my friends”), and support from significant others (4 items; e.g., “there is a special person who is around when I am in need”). Extant literature has shown that compared with seven-point formats (based on which the original MSPSS was developed among North American students), responses from East Asian (especially Chinese) students using five-point scales are less biased (Chen et al., 1995; Ares, 2018; de Rezende and de Medeiros, 2022; Yichen and Chuntian, 2024). Also, in order to avoid having two separate instructions and potentially confusing students in the second survey, a five-point rating scale for the MSPSS ranging from 1 to 5 was adopted according to a prior study conducted among Chinese university students (Yichen and Chuntian, 2024). In the current study, this scale showed good model fit (χ^2^/df = 1.524, CFI = 0.984, TLI = 0.980, and RMSEA = 0.059) and the McDonald's ω of the whole instrument, support from family subscale, support from friends subscale, and support from significant others subscale was 0.957, 0.918, 0.932, and 0.930, respectively.

The Chinese version of the Schwartz Outcome Scale (SOS-10) adopted from Fu et al. (2024) was utilized to evaluate university students' overall psychological adjustment. Originally developed by Blais et al. (1999), this is a unidimensional measure of 10 items (e.g., I feel hopeful about my future). In the present study, this model revealed sound fit to the data (χ^2^/df = 3.327, CFI = 0.954, TLI = 0.935, and RMSEA = 0.083) and the ω was 0.900.

### 2.3 Data analysis

Data in the present study were analyzed with SPSS 26.0, Mplus 8.3, and AMOS 23.0. SPSS was utilized for descriptive statistics, reliability analyses, correlation analyses, and regression analysis. Mplus was used to assess the structural validity and measurement equivalence of the ISSS-C in the first survey through maximum likelihood with robust standard errors (MLR) method. AMOS was used to test model fit of scales in the second and the third survey via maximum likelihood (ML) method. Specifically, first, items in the original ISSS were examined to ensure they were normally distributed and connected with their corresponding subscales. Second, confirmatory factor analyses (CFA) were carried out to assess the factorial structure of the ISSS-C. Third, reliability of the instrument and its dimensions was evaluated using McDonald's omega coefficients. Forth, the ISSS-C was further tested through multi-group confirmatory factor analysis (MGCFA) to ensure its measurement equivalence across student groups from different sizes of hometown. Next, Bivariate correlation analyses between the two ISSS-C dimensions and the three dimensions of the MSPSS Chinese version were conducted to confirm the criterion validity of the new instrument. Lastly, a multivariate regression analysis was performed with the two ISSS-C factors as independent variables and psychological adjustment as dependent variable to test if social support in intra-country migration settings can predict psychological adjustment, and also attempted to establish incremental validity for the ISSS-C. The significance level of *p* < 0.001, *p* < 0.01, and *p* < 0.05 were chosen for all analyses.

## 3 Results

### 3.1 Item analysis

First, data normality of all ISSS items were assessed through Kolmogorov-Smirnov tests. The results showed that items were not normally distributed in strict terms (*ps* < 0.05). The skewness and kurtosis values of every item in the ISSS were also inspected, which all lay between +2 and −2, indicating the data of every item still generally followed normal distribution (Tabachnick and Fidell, 2007). Then, polychoric correlation analyses were carried out between every item and the mean of the dimension it belonged. Results revealed that they were significantly correlated (with polychoric correlation coefficients ranging from 0.596 to 0.717). Hence, all the items were retained for further analyses.

### 3.2 Confirmatory factor analysis

CFA was performed. The results revealed that standard loadings of items in the socio-emotional support dimension was 0.79, 0.85, 0.84, 0.87, 0.87, 0.90, 0.83, 0.84, and 0.86, respectively, and those in the instrumental support dimension was 0.84, 0.82, 0.86, 0.89, 0.83, 0.88, 0.88, 0.80, and 0.82, all exceeding 0.50. The minimum, maximum, and median of all items were 1, 5, and 3. According to Schmitt (2011), the model fit was excellent (χ^2^/df = 5.641, CFI = 0.955, TLI = 0.949, SRMR = 0.028, and RMSEA = 0.062). The composite reliability (CR) of the two factors were 0.959 and 0.958, and the average variance extracted (AVE) of them were 0.721 and 0.716. Both CR and AVE values were above their respective thresholds of 0.60 and 0.50 (Fornell and Larcker, 1981), confirming convergent validity. The discriminant validity was determined via the heterotrait-monotrait ratio of correlations (HTMT). The result indicated that the HTMT ratio between the two factors was 0.868, lower than the threshold of 0.90 (Henseler et al., 2015), which confirmed the attainment of discriminant validity. Therefore, the two-factor ISSS Chinese version (the ISSS-C) exhibited good structural validity.

### 3.3 Reliability

The McDonald's omega values were used to determine the reliability of ISSS-C. The ω of the socio-emotional support dimension was 0.959 and that of the instrumental support dimension was 0.958. Hence, ISSS-C was tested to reveal high reliability.

### 3.4 Measurement equivalence across hometown sizes

In order to test the invariance of ISSS-C across participants' different hometown sizes, MGCFA were performed to investigate the configural invariance, the metric invariance, and the scalar invariance (Brown et al., 2015). The three groups compared were students from big cities (M_age_ = 19.6; 55.3% female), those from medium or small cities (M_age_ = 20.6; 46.0% female), and students from towns or villages (M_age_ = 20.5; 43.3% female). The results showed that the model fit of these three models were sound and the Δ CFI, Δ RMSEA, and Δ SRMR between them were all smaller than 0.01 (Table 2), suggesting that the measurement equivalence of ISSS-C across student groups from different types of hometown was valid.

**Table 2:** Measurement equivalence across hometown sizes.

	**RMSEA**	**SRMR**	**CFI**	**Model compare**	**Δ RMSEA**	**Δ SRMR**	**Δ CFI**	**Decision**
M1: configural invariance	0.066 (0.062–0.071)	0.034	0.951					
M2: metric invariance	0.065 (0.061–0.070)	0.038	0.949	M1	0.001	0.004	0.002	Accept (Δ < 0.01)
M3: scalar invariance	0.064 (0.060–0.068)	0.039	0.947	M2	0.001	0.001	0.002	Accept (Δ < 0.01)

### 3.5 Criterion validity

To test the criterion validity of the ISSS-C, criterion variables theoretically relevant to social support in intercultural contexts were used. In the current study, the three dimensions (support from family, support from friends, and support from significant others) of the Chinese version of the MSPSS were adopted as criterion variables. The data collected in the second survey (*n* = 151) were used to perform this test. The ISSS-C model exhibited acceptable fit to the data (χ^2^/df = 2.947, CFI = 0.911, TLI = 0.898, SRMR = 0.045, and RMSEA = 0.114). Bivariate correlation analyses between the two dimensions of the ISSS-C and the three Chinese Version MSPSS dimensions were conducted. As shown in Table 3, socio-emotional support and instrumental support were positively correlated with all three criterion variables (*p* < 0.01), thus supporting criterion validity.

**Table 3:** Correlation coefficients of the ISSS-C and criterion variables.

**Criterion variables**	**Socio-emotional support**	**Instrumental support**
Support from significant others	0.692^**^	0.710^**^
Support from family	0.612^**^	0.640^**^
Support from friends	0.610^**^	0.682^**^

^**^p < 0.01.

### 3.6 Incremental validity

As suggested by Ong and Ward (2005) who developed the original ISSS, incremental validity of the scale can be tested by assessing the predictive effects of the scale's two factors on psychological adjustment. This analysis used the data set obtained from the third survey (*n* = 335). The ISSS-C also showed acceptable model fit (χ^2^/df = 3.766, CFI = 0.942, TLI = 0.934, SRMR = 0.040, and RMSEA = 0.091). Before the regression analysis, tests were first performed to ensure the data were normally distributed and there were no multicollinearity issues. The skewness and kurtosis values of all items in the ISSS were examined, which indicates the data of every item generally followed normal distribution (Tabachnick and Fidell, 2007). The existence of multicollinearity was detected with three values: tolerance, variance inflation factor (VIF), and condition index (CI). The results showed that tolerance, VIF, and CI of all variables in the data fell within the threshold of 0.10, 10, and 30 (Hair et al., 2010; Sekaran and Bougie, 2010). Thus, the data set did not have multicollinearity problems. Then, a multivariate regression analysis was carried out on the sample obtained from the third survey with socio-emotional support and instrumental support as independent variables and psychological adjustment (measured by the Chinese version of the SOS-10) as the dependent variable. The results showed that instrumental support had a direct contribution to psychological adjustment (β = 0.467, *t* = 6.816, *p* < 0.05), but socio-emotional support did not influence psychological adjustment (β = 0.127, *t* = 1.845, *p* > 0.05). Such findings are in consistent with Ong and Ward (2005)'s original study, establishing incremental validity for instrumental support but not socio-emotional support.

## 4 Discussion

Social support is a meta-construct conceptualized distinctly depending on different purposes or situations in operation (Vaux, 1988; Vazquez-Morejon and Garcia-Boveda, 1997). Thus, cross-cultural studies using instruments not specifically designed for such purposes could produce invalid results (Veiel, 1985; Ong and Ward, 2005). Social support in cross-cultural contexts typically comprises socio-emotional support and instrumental support, and is known to alleviate stress and facilitate psychological adjustment to distinct cultural conditions (e.g., Adelman, 1988; Walton, 1990; Zhang and Goodson, 2011; English et al., 2021). However, for a long period, no social support instrument tailor-made for intercultural studies existed until the development of the Index of Sojourner Social Support by Ong and Ward (2005). Since then this scale has been widely applied in different countries, including China. Nevertheless, despite its popularity in China, particularly in internal migration conditions, no previous study has thoroughly validated the ISSS in Chinese contexts, potentially affecting the validity of studies using this scale, given the fact that the dimensionality of scales related to intercultural issues often vary across cultures (Bahar-Özvarıs et al., 2022; Bücker et al., 2015). It is, therefore, highly necessary to develop a validated Chinese version of the ISSS.

In the current study, items in the original ISSS were first translated from English into Chinese and then back-translated into English by local and native English-speaking teachers according to strict procedures (Brislin, 1980), in order to ensure the semantic consistency between the original and the Chinese version. Next, item analysis showed that the data of all the items were normally distributed in general and related to their corresponding dimensions. CFA that followed confirmed the sound model fit, convergent validity, discriminant validity, and structural validity for this Chinese version scale. Reliability assessment based on McDonald's ω tests indicated the ISSS-C's good reliability. The results of the measurement equivalence test using multi-group confirmatory factor analysis further showed the ISSS-C's equal measurement significance across student groups of different hometown sizes.

Criterion validity was then confirmed by bivariate correlation analyses between two ISSS-C dimensions and three theoretically relevant variables (support from family, support from friends, and support from significant others) from another widely-used scale that measures general perceived social support—the Chinese version of the MSPSS. Results showed that the two dimensions of the ISSS-C were positively correlated with the latter three variables (*p* < 0.01), indicating that the ISSS-C has sound criterion validity. It further revealed that support from family, friends, and other important individuals plays essential roles in students' relocation to new cultural environments within China.

Lastly, a regression analysis was performed to examine whether the two components of the ISSS-C can predict psychological adjustment in internal migration contexts and also test the incremental validity of the ISSS-C. The results revealed that instrumental support was predictive of university students' psychological adjustment, but socio-emotional support did not have a significant effect on psychological adjustment. Such findings were in agreement with the prior study in inter-country migration contexts (Ong and Ward, 2005), which means providing informational assistance for mobile university students in intra-country migration settings is as vital as that in inter-country ones, and, similar to the original ISSS, incremental validity of the ISSS-C in internal migration conditions was also partially established.

The findings of the current study indicates that, in internal migration settings in China, perceived social support also comprises two facets, demonstrating conceptual alignment with the original constructs (Ong and Ward, 2005). Unlike the previous mixed results of studies attempting to validate the ISSS in other non-English speaking contexts (Gilbert and Rhodes, 2012; Rhodes et al., 2013), this newly adapted and validated scale (the ISSS-C) consists of the same 18 items. It can be a useful tool for assessing the levels of socio-emotional and instrumental support accessible to internal migrant populations in future research. Furthermore, the findings of the current study also underscore the importance of instrumental support in contributing to individuals' psychological adjustment in domestic migration contexts.

Prior studies have found that university students who fail to psychologically adjust well to new cultural environments often suffer from depression, anxiety, loneliness, and academic difficulties (Nadeem et al., 2020; Corradi and Levrau, 2021). In order to help students overcome these challenges as well as improve their emotional wellbeing, satisfaction with life, and a sense of purpose and hope through enhancing psychological adjustment (Ward and Kennedy, 1999), the findings of the current study suggest that, for university administrators and local communities in Shanghai, providing relevant students with informational (rather than emotional) assistance can be more effective. Further, the approaches mentioned in the ISSS-C, such as helping students with local institutions' official rules and regulations, can be an ideal guide for local university administrators and communities to consider which specific types of instrumental support should be provided.

Taken together, the ISSS-C has the same two dimensions as the original ISSS. It also exhibits excellent reliability and validity. Nonetheless, this study still has a number of limitations. First, to ensure the generalizablity and stability of this instrument in Chinese contexts, further tests among other populations apart from university students still need to be conducted (Cheng et al., 2020). Second, this study adopted a cross-sectional design. Future longitudinal studies may further test the reliability of this scale (Cheng et al., 2020). Third, the results could be affected by social desirability biases common in self-report surveys. Other methods assessing social support in internal migration settings could be considered in the future development of this line of research to supplement questionnaire surveys (Nadeem et al., 2023). Forth, the current study only explored the impact of the scale's two factors on psychological adjustment. Other relevant variables such as stress can be considered in future research (English and Worlton, 2017).

## 5 Conclusion

This study provides a validated and highly useful tool for measuring intercultural social support in cultural contexts of China—the ISSS-C. The results of this study indicate that the new scale has excellent psychometric properties and one of its dimensions—instrumental support—can assist Chinese university students in their psychological adjustment to local cultural conditions in their internal migrations.

## Data Availability

The raw data supporting the conclusions of this article will be made available by the author, without undue reservation.
